# Calcium dobesilate alleviates renal dysfunction and inflammation by targeting nuclear factor kappa B (NF-κB) signaling in sepsis-associated acute kidney injury

**DOI:** 10.1080/21655979.2021.2024394

**Published:** 2022-01-18

**Authors:** Zhijuan Xie, Lanji Wei, Jianying Chen, Zhong Chen

**Affiliations:** a Department of Nephrology, The First Affiliated Hospital of University of South China, Hengyang, Hunan, China; b Department of Health Management Center, Affiliated Nanhua Hospital, University of South China, Hengyang, Hunan, China; c Department of Rheumatology and Immunology, Hunan Provincial People’s Hospital, Changsha, Hunan, China; d Department of Nuclear Medicine, The First Affiliated Hospital of University of South China, Hengyang, Hunan, China

**Keywords:** Calcium dobesilate, NF-κB signaling, sepsis, acute kidney injury

## Abstract

Acute kidney injury (AKI) is a serious complication of sepsis that increases mortality and the risk of progression to chronic kidney disease. Oxidative stress and apoptosis are reported to exert critical function in the pathogenesis of sepsis-associated AKI. Calcium dobesilate (CaD) was reported to play a protective role in renal diseases. Therefore, we explored the antioxidant effect and potential mechanism of CaD in lipopolysaccharide (LPS)-induced AKI in mice. We evaluated renal function (blood urea nitrogen (BUN) and serum creatinine (SCr)), histopathology, oxidative stress (superoxide dismutase (SOD) and malondialdehyde (MDA)), inflammation cytokines, and apoptosis in kidneys of mice. The effect of CaD on NF-κB signaling was evaluated by Western blot. Our findings showed that CaD alleviated renal dysfunction and kidney injury, and also reversed upregulated MDA concentration and reduced SOD enzyme activity in AKI mice. Moreover, LPS-induced inflammatory response was attenuated by CaD. CaD treatment also reduced the apoptosis evoked by LPS. Additionally, CaD downregulated phosphorylation of nuclear factor kappa B (NF-κB) signaling components in LPS mice. Conclusively, CaD alleviates renal dysfunction and inflammation by targeting NF-κB signaling in sepsis-associated AKI.

## GRAPHICAL ABSTRACT

**Figure:**
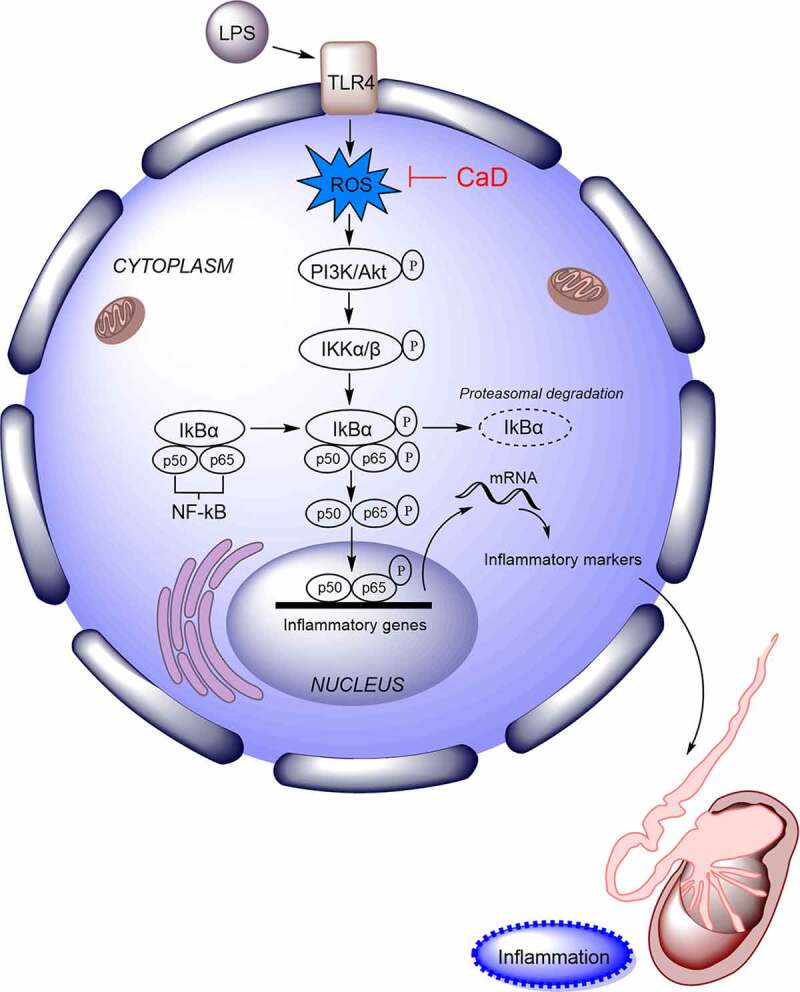

## Introduction

Sepsis has been known as a systemic inflammatory response syndrome which is induced by fungi, viruses, or bacteria. Importantly, sepsis can result in shock, dysfunction syndrome of multiple organs, and even death [1]. In the development of sepsis, acute kidney injury (AKI) is one of the complications which is common and serious. Increasing research has shown that sepsis facilitates the inflammatory response in renal tissue, causing significant apoptosis of renal cells, thereby leading to AKI [2–4]. Previously, functional abnormalities and dysregulation of various genes, RNA and proteins have been identified during AKI progression, and effectively regulating expression of these abnormal molecules may affect AKI [5–7]. Therefore, an in-depth investigation of novel therapeutic targets is desirable to improve patient outcome and may optimize AKI treatment in the future.

Calcium dobesilate (CaD) is an angio-protective agent which is widely used to normalize vascular dysfunction in diverse diseases over 40 years, especially in diabetic retinopathy [8,9], diabetic nephropathy [10,11], as conferring possible benefit but not giving rise to side effects [12]. In a study concerning clinical evaluation of the efficacy and safety of CaD in patients with chronic venous insufficiency of the lower limbs, 22 side effects were reported in 63 patients (17.9%) at some time during treatment, the most important including headache, gastralgia, and dizziness, which is an acceptable safety profile [13]. CaD exhibits as an antioxidant property with microcirculation alteration and without synthesis action [14–16]. It can also improve vascular permeability and inhibit the synthesis of collagen in basement membrane in diabetic mice [17,18]. Due to strong antioxidant property, CaD possibly prevents alterations in concentrations of creatinine and urea nitrogen [19,20]. Importantly, evidence has indicated that nuclear factor-kappa B (NF-κB) pathway activation induced by high glucose in diabetic retinopathy is inhibited by CaD treatment [21]. However, studies concerning CaD effectiveness in sepsis-induced AKI have not been available. Therefore, it is urgent to elucidate pathogenic mechanisms underlying sepsis-induced AKI to improve therapies for this disease.

NF-κB signaling pathway has been extensively reported to modulate sepsis-induced AKI [22–24]. During the activation of NF-κB signaling, the IκBKinase (IKK) complex was activated, and IκB was phosphorylated. The phosphorylation of IκB results in its ubiquitination and proteasomal degradation, releasing NF-κB/Rel complexes [25,26]. Thus, NF-κB, a transcription factor, is released, translocated into nucleus, and then activates the transcription of cytokines and antiapoptotic genes [25,26]. In addition, toll-like receptor 4 (TLR4) and p65 exert significant functions in sepsis-induced AKI [25–27].

In this research, we hypothesized that CaD has the renal protective effects in sepsis-induced AKI via mediating NF-κB signaling. We investigated the effects of CaD on kidney injury, oxidative stress, inflammatory response, and apoptosis in an AKI mouse model using lipopolysaccharide (LPS). Our research may provide promising information for a more effective approach for enhancing chemo-protection against sepsis-induced AKI.

## Materials and methods

### Animals

A total of 30 male C57BL/6 mice weighing 20–22 g were purchased from Vital River Co. Ltd., Beijing, China. Animals were housed in standard conditions, and randomly divided into three groups. Mice in the lipopolysaccharide (LPS) group were intraperitoneally injected with LPS (10 mg/kg; Sigma-Aldrich, St. Louis, CA, USA). Mice in the sham group were intraperitoneally injected with the same dose of saline solution. CaD (purity: 99%) was purchased from Henan DaKen Chemical CO., LTD. (cat. 21009; Henan, China). Mice in the LPS + CaD group received intragastric administration of CaD (100 mg/kg/day) for three consecutive days before LPS injection [11,28]. Subsequently, survival rate was recorded after LPS administration for the next 120 h. All experimental procedures were approved by the Ethics Committee of The First Affiliated Hospital of University of South China (Hunan, China; approval number: 2021ll1221001).

### Kidney function test

Blood samples were collected from the inferior caval vein. After centrifugation at 2,000 g for 20 min, the serum was separated and kept at −80°C until being assayed for blood urea nitrogen (BUN) and serum creatinine (Scr) [29]. The biochemical parameter levels were evaluated using an automatic cell analyzer (Abbott Corporation, Chicago, USA).

### Histological analysis

The kidney tissues were fixed in 10% formalin and were embedded in paraffin. Sections of the tissue were cut into 5 μm and mounted on slides. Following drying at 37°C, deparaffinization and rehydration through a series of xylene and alcohol solutions, sections were subjected to hematoxylin–eosin and periodic acid–Schiff staining under a light microscope (Nikon Corporation, Tokyo, Japan) [30]. The degree of acute tubular injury was scored by the loss of brush border, tubular dilation, cast formation, and cell lysis. In each kidney tissue, five different areas were assessed and scored based on the following standard: 0, normal histology; 1, 25% of tubules showed pathological damage; 2, 25–50% damage; 3, 50–75% damage; 4, >75% damage [31].

### Oxidative stress evaluation

The level of malondialdehyde (MDA) in the tissue homogenates was taken as the level of lipid oxidation in the tissue, while the superoxide dismutase (SOD) was the index of the tissue antioxidant. The levels of MDA and the activities of SOD were detected utilizing commercial detection kits (Nanjing KeyGen Biotech. Co. Ltd., Nanjing, China) following the manufacturer’s protocols [32].

### Real-time quantitative polymerase chain reaction (RT-qPCR)

As described previously [33], extraction of total RNA from kidney tissue was carried out. Using RevertAid First-Strand cDNA Synthesis kits (Thermo Scientific, Waltham, USA), the complementary DNA (cDNA) was synthesized. On CFX96 Touch™ Deep Well Real-Time PCR Detection System (Bio-Rad, Hercules, CA, USA), Synergy Brands (SYBR) Green (Bio-Rad) was used to perform quantitative real-time PCR for detecting expression of mRNA for inducible nitric oxide synthase (iNOS). The 2^−ΔΔCt^ method was used to process data.

### Enzyme-linked immunosorbent assay (ELISA)

The serum of LPS-treated mice was collected using blood collection tubes, centrifuged at 2,000 g for 20 min, and the supernatant was collected [34]. The levels of interleukin-6 (IL-8), IL-6, interleukin-1β (IL-1β), and tumor necrosis factor-alpha (TNF-α) were measured using ELISA kits (Beyotime, Shanghai, China) according to the manufacturer’s instructions.

### Terminal-deoxynucleotidyl transferase-mediated nick end labeling (TUNEL) analysis

TUNEL assay was conducted to examine apoptotic cells in the renal tissue using the In Situ Cell Death Detection kit (Roche Molecular Biochemicals, Mannheim, Germany) according to the manufacturer’s instructions. As previously described [35], the kidney sections were hydrated in phosphate buffer saline and subsequently immersed in permeabilization solution (0.1% Triton X-100) for 3 min. Next, 4′,6-diamidino-2-phenylindole (DAPI) was used to stain nuclei after TUNEL reaction. The apoptotic ratio was expressed as the number of positive cells to total cells.

### Immunofluorescence staining

The kidney tissues were cut into 5-μm-thick slices on a cryostat and stored at −20°C until use [36]. The tissue sections were blocked with 1% bull serum albumin for 1 h at room temperature, and then incubated overnight with phospho-NF-κB p65 rabbit mAb (Cell Signaling Technology, Danvers, MA, USA) followed by incubation with Alexa Fluor 488-conjugated goat anti-rabbit IgG (Abcam, Cambridge, UK). The DAPI staining was conducted according to the experimental protocol. Morphological analyses were performed by using AX10 imager A2/AX10 cam HRC fluorescence microscope (Zeiss, Jena, Germany).

### Western blot

Western blot was performed as previously published [37]. Total protein from tissues was extracted using radioimmunoprecipitation assay lysis buffer (Sangon Biotech, Shanghai, China) containing 1% Ethylene Diamine Tetraacetic Acid solution and 1% protease inhibitor cocktails (Sigma-Aldrich). The bicinchoninic acid assay kit (Beyotime) was used to evaluate protein concentration. Using electrophoresis, the isolated proteins were transferred onto polyvinylidene difluoride membranes. Next, the proteins were blocked with 5% skimmed milk for 1 h, and then incubated with primary antibodies including Bax (ab32502, Abcam), Bcl-2 (ab182858), p-PI3K (#17366, Cell Signaling Technology), PI3K (#4249), p-AKT (#4060), AKT (#4685), p-IKKβ (#2078), IKKβ (#8943), p-IKBα (ab133462), IKBα (ab32518), p-p65 (ab239882), p65 (ab207297), GAPDH (ab181602), and Histone H3 (ab1791) overnight at 4°C. The next day, the proteins were incubated with horseradish peroxidase-conjugated goat anti-rabbit IgG in Tris-buffered saline (TBS) for 2 h at room temperature. The Enhanced Chemiluminescent kits (GE Healthcare Bio-Sciences, Pittsburgh, PA, USA) were utilized to visualize the proteins. Protein bands were detected using Image Lab Touch 3.0 Software (Bio-Rad, Hercules, USA).

### Statistical analysis

The analysis was conducted utilizing SPSS version 23 (SPSS Inc., Chicago, IL, USA). Data are shown as mean ± standard error of the mean (SEM). Statistical analysis was carried out by one-way analysis of variance (ANOVA) or Student’s t-test, and then Bonferroni analysis was performed. The value of *p* < 0.05 was considered statistically significant.

## Results

In this study, we established an AKI mouse model using LPS to explore the function of CaD on kidney injury, oxidative stress, inflammatory response, and apoptosis. We hypothesized that CaD has a renal protective role against LPS-induced AKI. Our results showed that depletion of CaD pre-treatment alleviated renal dysfunction and kidney injury, and attenuated oxidative stress, inflammatory response, and apoptosis in LPS mice. Additionally, CaD inhibited activation of NF-κB signaling LPS mice. Overall, CaD alleviates renal dysfunction and inflammation by targeting NF-κB signaling in sepsis-associated AKI.

### CaD improved mouse survival and alleviated renal dysfunction in LPS mice

**Figure 1.**
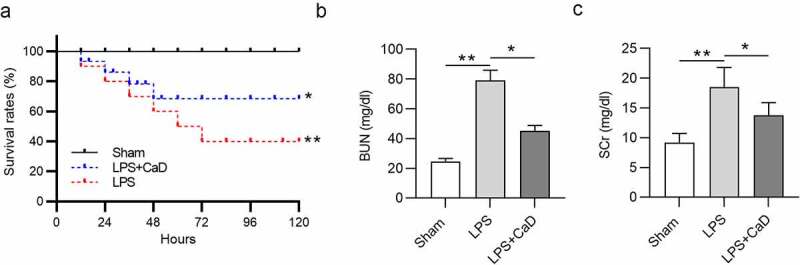
**Effect of CaD on survival and renal dysfunction**. (a) Survival rate of mice in sham, LPS and LPS + CaD groups. (b-c) BUN and SCr levels in kidneys in mice in sham, LPS and LPS + CaD groups. N = 10 in each group. *p  <  0.05; **p  <  0.01.

As shown in Figure 1a, after LPS injection, the survival rate of mice was decreased in a time-dependent manner: 80% at 24 h, 60% at 48 h, and 40% in 2 days (*p* < 0.01). However, CaD treatment significantly increased survival rate and extended lifetime compared to the LPS group (*p* < 0.05). BUN and SCr levels were detected to evaluate kidney function. Compared to the sham group, BUN and SCr levels were found to be significantly high in the LPS group (*p* < 0.01), while CaD treatment reduced BUN and SCr levels in mice treated with LPS (*p* < 0.05) (Figure 1(b,c)).

### CaD restored pathological changes in LPS mice

**Figure 2.**
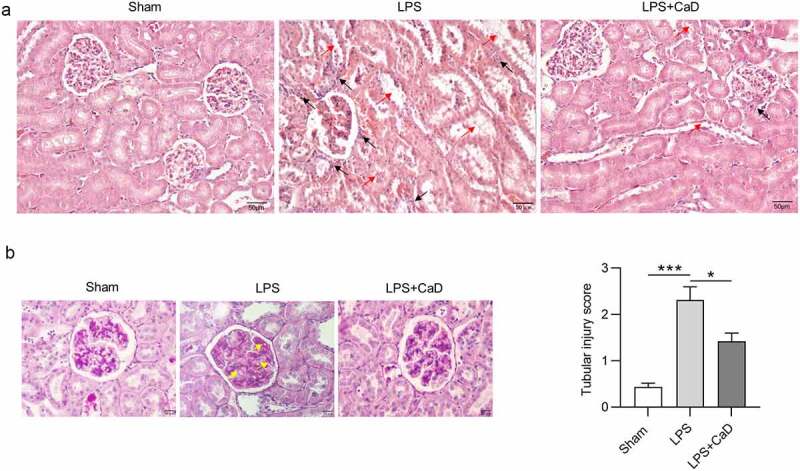
**LPS-induced AKI was alleviated by CaD**. (a) Hematoxylin and eosin staining of kidney sections in various groups. (b) Periodic acid Schiff staining of kidney sections in various groups. (c) Tubular injury score of kidney sections in various groups. Red arrow presents severe granular in renal tubular epithelial cells, vacuolar degeneration, and renal tubular necrosis. Black arrow presents inflammatory cellular infiltration in the renal interstitium. Yellow arrow presents thickening of the mesangial and basement membrane. N = 10 in each group. *p  <  0.05; ***p  <  0.001.

In addition, according to hematoxylin and eosin staining assays (Figure 2a), we found the alteration in mesangium expansion, vascular congestion, tubular dilatation, hypercellularity, tubular necrosis, and degeneration in LPS mice. Administration of CaD restored these pathological changes as compared to untreated mice. Additionally, inflammatory cellular infiltration was significantly increased in the renal interstitium after LPS treatment. These histological changes were attenuated by CaD pre-treatment in LPS-subjected mice. Furthermore, periodic acid Schiff staining of LPS-challenged kidneys revealed destruction of tubular brush border and tubular vacuolization as indicated by Figure 2b. All of these alterations were alleviated in the mice which were treated with CaD. Tubular injury score in the LPS group was higher than in the sham group (*p* < 0.001), while CaD pre-treatment significantly decreased the injury score (*p* < 0.01) (Figure 2c).

### CaD alleviated oxidative stress and nitrosative stress

**Figure 3.**
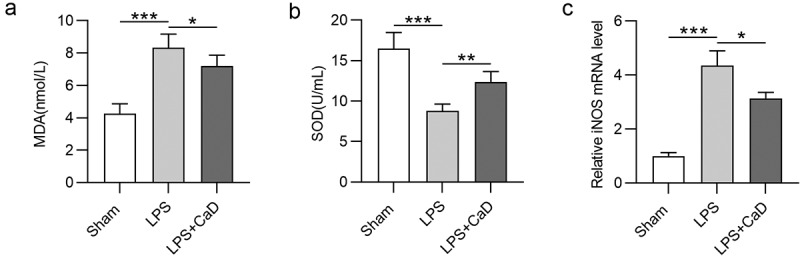
**Effect of CaD on oxidative stress and nitrosative stress in LPS-induced AKI**. (a-b) MDA and SOD levels in kidneys of sham and experimental groups of mice were determine. (c) The mRNA level for iNOS was detected by RT-qPCR. N = 10 in each group. *p  <  0.05; **p  <  0.01; ***p  <  0.001.

It has been reported that LPS-induced AKI destroys the balance of intracellular redox [16]. As shown in Figure 3(a,b), MDA concentration was upregulated, and SOD enzyme activity concentration was reduced following LPS-induced AKI in LPS group compared to the sham group (*p* < 0.001). These levels were markedly reversed due to CaD administration in LPS mice (*p* < 0.01). Additionally, the mRNA level for iNOS was upregulated in kidneys of LPS mice compared to the sham group (*p* < 0.001). Cad pre-treatment decreased the iNOS level in LPS mice as compared to untreated mice (*p* < 0.05) (Figure 3c).

### CaD inhibited the inflammatory response

**Figure 4.**
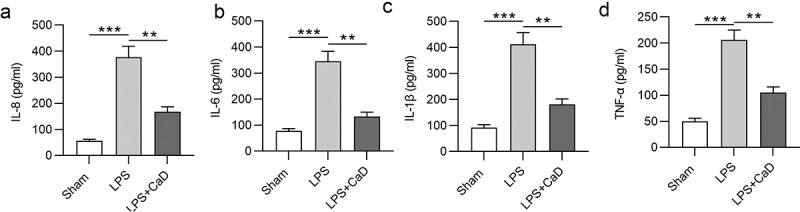
**Inflammatory response was inhibited CaD treatment**. Renal inflammatory marker levels of (a) IL-8, (b) IL-6, (c) IL-1β, and (d) TNF-α in various experimental groups were determined using ELISA. N = 10 in each group. **p  <  0.01; ***p  <  0.001.

Next, the effect of CaD on the inflammatory response was analyzed by measuring cytokine (IL-8, IL-6, IL-1β, and TNF-α) concentration in the indicated group. The results of ELISA showed that the levels of IL-8, IL-6, IL-1β, and TNF-α in serum of LPS mice were upregulated compared to the sham group (*p* < 0.001), whereas these upregulated levels were reversed in the LPS + Cad group (*p* < 0.01) (Figure 4(a–d)).

### CaD reduced apoptosis in LPS mice

**Figure 5.**
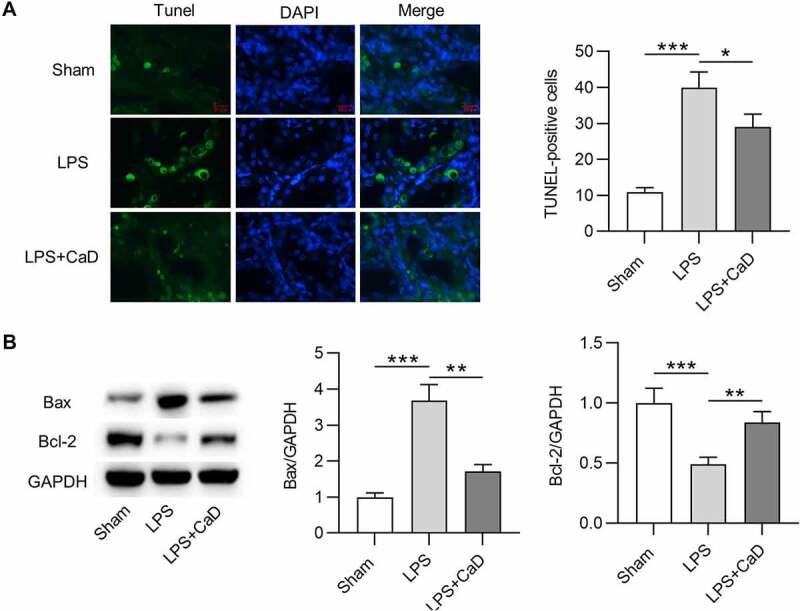
**The apoptosis in mouse model of AKI was reduced after CaD treatment**. (a) TUNEL staining of kidney sections in various groups and quantitation of TUNEL-positive cells. (b) Western blot analysis of kidney sections in various groups and quantitation of Bax and Bcl-2 protein levels. N = 10 in each group. *p  <  0.05; **p  <  0.01; ***p  <  0.001.

In this section, TUNEL assay and Western blot analysis were conducted to evaluate the effect of LPS and CaD on cell apoptosis. Compared to mice in the sham group, the number of TUNEL-positive cells was higher in LPS mice (*p* < 0.001), and the mice in the LPS + CaD group showed fewer positive cells than LPS mice (*p* < 0.05) (Figure 5a). Furthermore, Western blot demonstrated that LPS caused an increase in Bax protein level and a decrease in Bcl-2 protein level in kidneys compared to the sham group (*p* < 0.001). However, Bax and Bcl-2 protein levels were significantly reversed due to CaD administration in LPS mice (*p* < 0.01) (Figure 5b).

### CaD restrained LPS-induced activation of NF-κB

**Figure 6.**
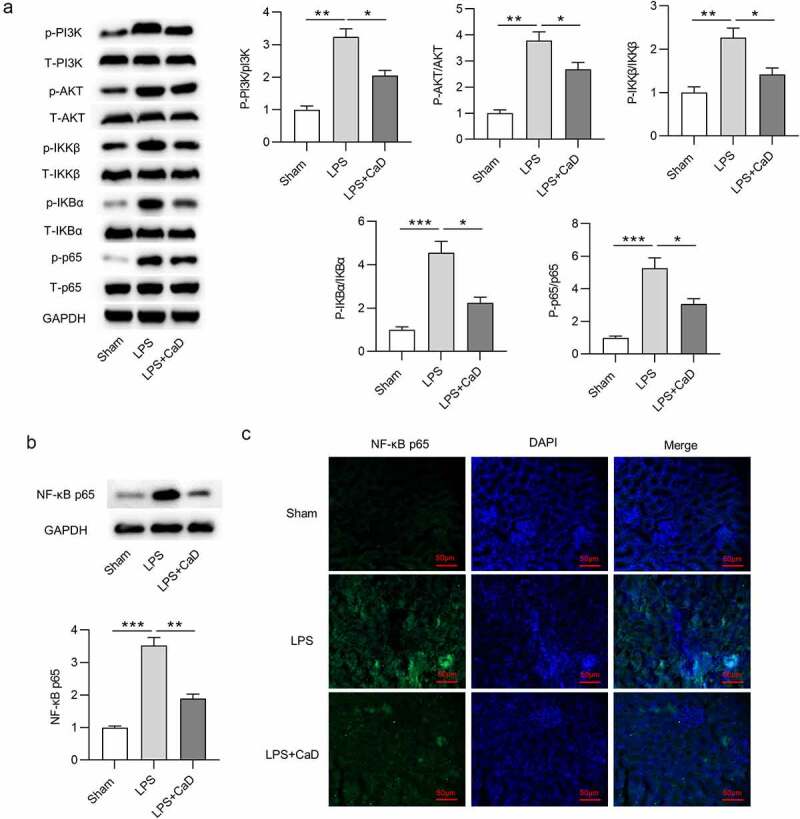
**The regulatory role of CaD in LPS-induced activation of NF-κB**. (a) The phosphorylation and total levels of PI3K, AKT, IKKβ, IKBα, p65 in kidney sections in various groups were detected by using Western blot analysis. (b) NF-κB p65 nuclear translocation levels in kidneys were determined using Western blot analysis. (c) Representative images showing the nuclear translocation of NF-κB p65 in kidneys. N = 10 in each group. *p  <  0.05; **p  <  0.01; ***p  <  0.001.

To determine whether NF-κB pathway is involved in the CaD-mediated suppression of AKI, NF-κB signaling pathway activity was assessed by testing the protein level of NF-κB signaling components. Western blot analysis revealed that CaD treatment inhibited phosphorylation of PI3K, AKT, IKKβ, IKBα, p65 in kidneys of LPS mice (*p* < 0.05) (Figure 6a). Meanwhile, Western blot showed that CaD significantly decreased the LPS-upregulated nuclear protein levels of NF-κB in kidneys (*p* < 0.01) (Figure 6b). Immunofluorescence staining further showed that CaD inhibited the LPS-promoted NF-κB nuclear translocation in kidneys (Figure 6c).

## Discussion

AKI is one of the most serious complications of sepsis and is a rapid renal dysfunction associated with inflammation and oxidative stress. Intraperitoneal injection of LPS to induce sepsis is a commonly used animal model. LPS is a classic toll-like receptor 4 (TLR4) agonist which can induce an immediate and robust inflammatory response, thus stimulating activation of the innate immune system in human sepsis [38]. The aim of the present study was to evaluate the potential effects of CaD, an angio-protective agent, on renal damage in mice with LPS-induced sepsis. Our results revealed that treatment with CaD significantly alleviated LPS-induced oxidative stress, inflammation, and apoptosis which consequently attenuated kidney dysfunction. The underlying molecular mechanism of CaD’s renal protective effects appeared to involve the suppression of NF-κB activation. Therefore, the results of the present study suggested that CaD potentially has beneficial effects in therapies to prevent sepsis-induced AKI.

The protective effects of CaD have been reported against oxidative stress, apoptosis, and pyroptosis in the kidney of burned mice [39]. Oxidative stress has been identified as one of the critical contributors of pathogenesis in AKI [40,41]. Production of free radicals is eliminated by intracellular antioxidant enzymes such as SOD [42], thus maintaining the balance between production and elimination of free radicals. AKI increases superoxide production and inhibits SOD activity. MDA is the end product of lipid peroxidation, and its production is increased in kidney tissue after renal ischemia reperfusion injury [42]; MDA contributes to cell apoptosis and is strongly involved in AKI [43]. Additionally, we demonstrated that CaD downregulated MDA levels and upregulated SOD levels. The kidney consists of tubules, renal vesicles, and glomeruli. Studies have reported that sepsis-related AKI is principally caused by glomerular or tubular apoptosis [44]. Our *in vivo* data demonstrated that CaD alleviated LPS-induced apoptosis in kidney tissue, suggesting that the protective effects of CaD in sepsis-related AKI are partially due to alleviation of oxidative stress and apoptosis.

We found that the levels of BUN and SCr were increased after LPS surgery; these findings are consistent with previous reports [45,46]. Our study showed that histological changes in kidney function after LPS treatment could be restored by CaD, since significant reduction in BUN and SCr levels were identified. Nitric oxide (NO) is an important proinflammatory molecule that is released during inflammatory response. In pathological conditions, iNOS is induced and then NO is synthesized, which can affect many parts of the inflammatory cascade [47]. There is substantial evidence showing that LPS-mediated renal inflammatory damage may be due to increased iNOS activity and consequent abnormal NO levels [48,49]. Our results showed that LPS-subjected mice had enhanced iNOS expression in the kidneys; these changes were significantly reduced by treatment with CaD.

TNF-α, IL-6, and IL-1β are involved in LPS-induced tissue damage and are regarded as major regulators of severe inflammatory diseases such as sepsis [50–52]. In this study, we found that CaD treatment reduced the levels of pro-inflammatory cytokines TNF-α, IL-6, IL-8, and IL-1β. Since the increased release of pro-inflammatory cytokines appears to be an essential aspect of pathogenesis in the inflammation process, the inhibitory effects of CaD on LPS-induced cytokine production might be a crucial step in the anti-inflammatory action of CaD. The nuclear transcription factor NF-κB amplifies and regulates many genes, including multiple cytokines and iNOS in response to inflammatory stimuli. When activated by such stimuli, NF-κB dissociates from IκB followed by translocation to the nucleus, leading to gene transcription [53]. NF-κB is a promising target for treating a variety of diseases because it plays a diverse role in the expression of inflammatory genes. In this study, CaD blocked the LPS-induced activation of NF-κB by inhibiting its translocation. These observations indicated that interference with NF-κB might explain, as least in part, the inhibitory effects of CaD on iNOS, TNF-α, IL-6, IL-8, and IL-1β levels.

## Conclusion

In conclusion, this study has demonstrated the renal protective effects by CaD against LPS-induced kidney injury. The ameliorative effects of CaD were associated with downregulation of pro-inflammatory cytokine production and reduction of iNOS expression by blocking NF-κB signaling pathway. These effects were accompanied by enhanced antioxidant defense in the kidney. Overall, our results suggest that CaD has the potential to be considered for therapeutic use in the treatment of renal inflammatory damage and sepsis-induced AKI. There are still limitations in this study. Future study endeavors are required to investigate the precise regulatory mechanisms of how CaD exerts its functions against LPS-induced kidney injury by NF-κB signaling. More importantly, it would be better to increase sample size for *in vivo* experiments to validate the results of our study.

## Supplementary Material

Supplemental Material
