# Supplementary figures and images for: Calcium dobesilate alleviates renal dysfunction and inflammation by targeting nuclear factor kappa B (NF-κB) signaling in sepsis-associated acute kidney injury
Source: Bioengineered. 2022 Jan 18;13(2):2816–26. doi: 10.1080/21655979.2021.2024394 (PMC8974157; doi:10.1080/21655979.2021.2024394)

Fig.5B

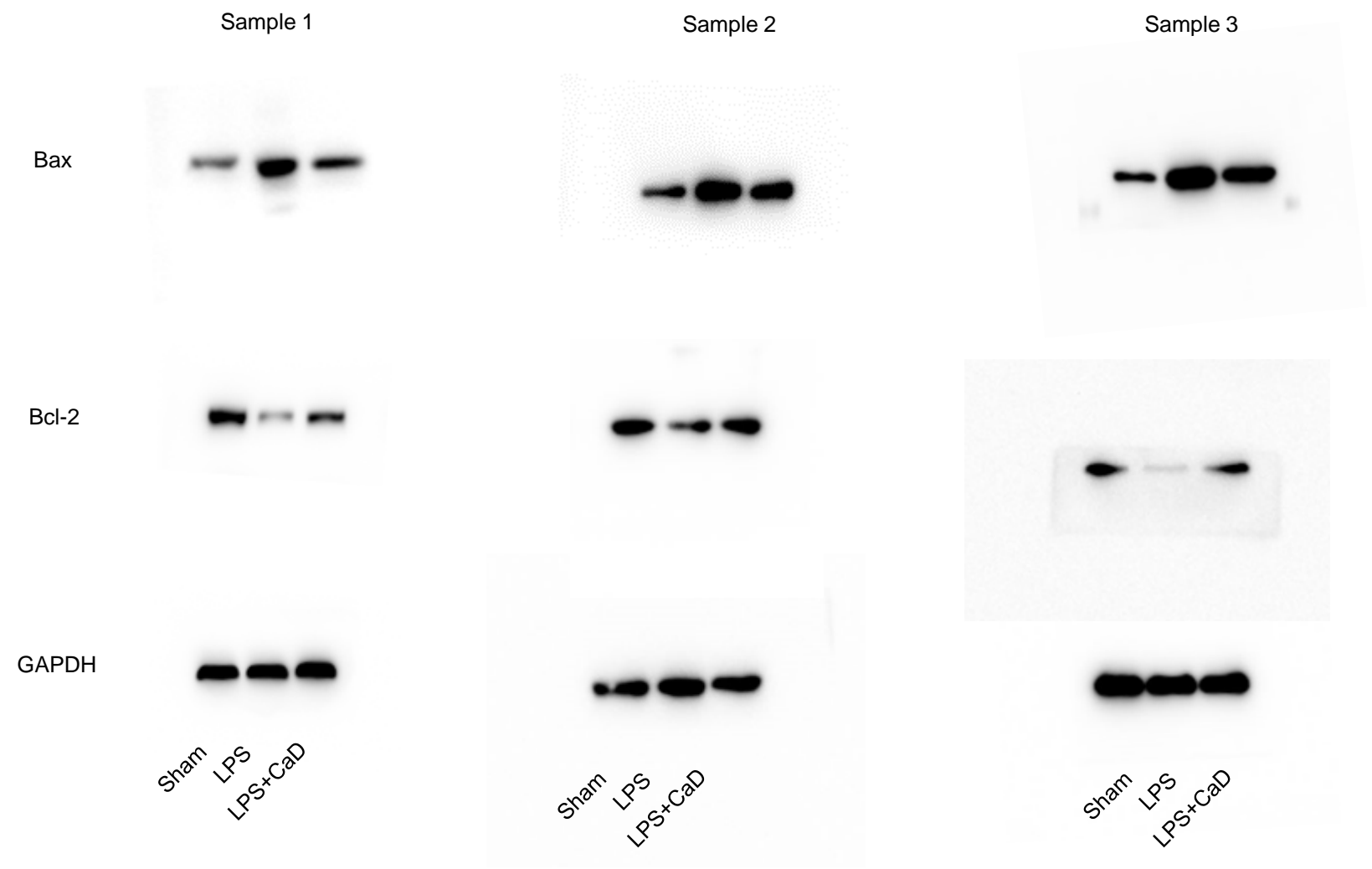

Fig.6A

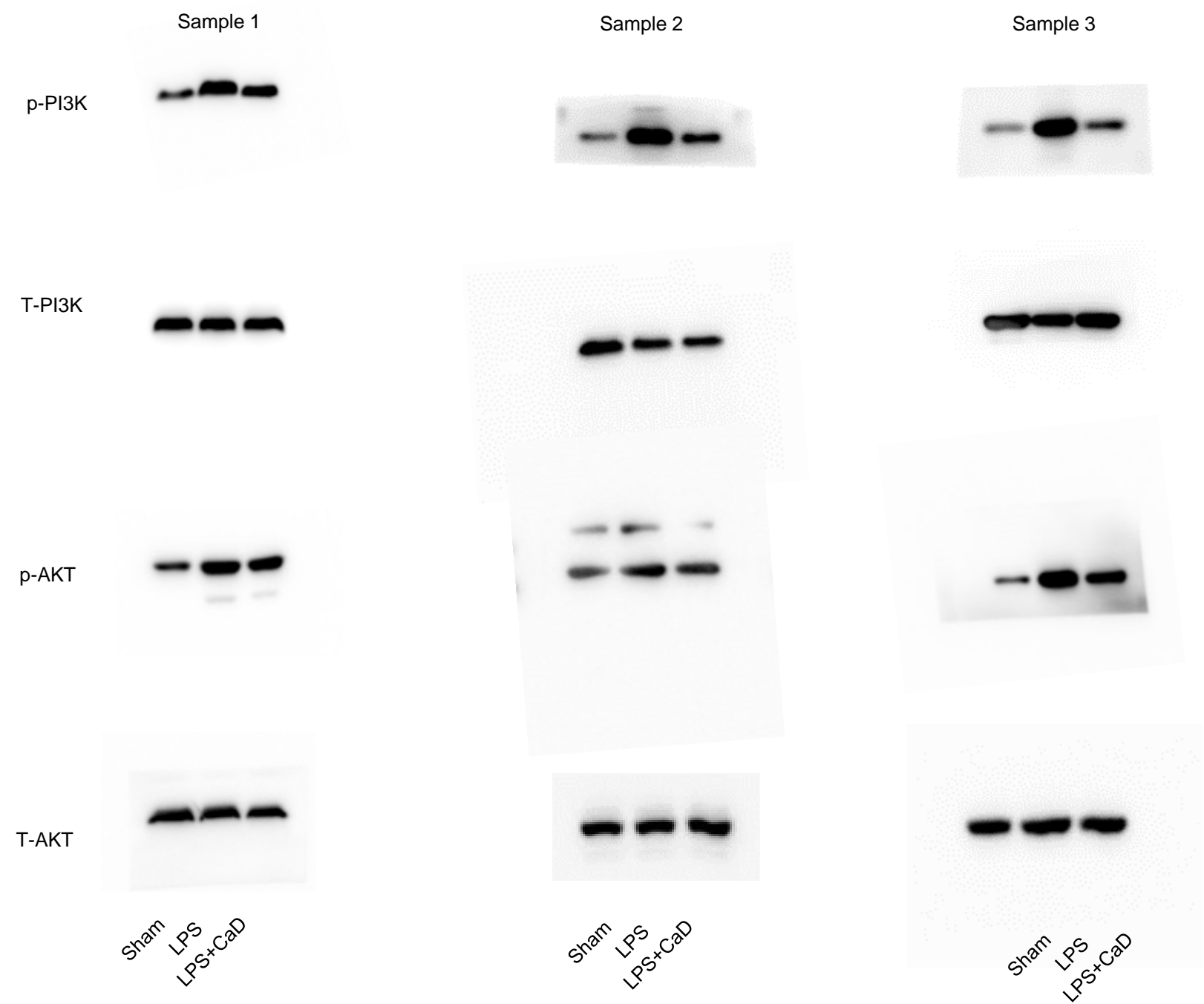

Fig.6A

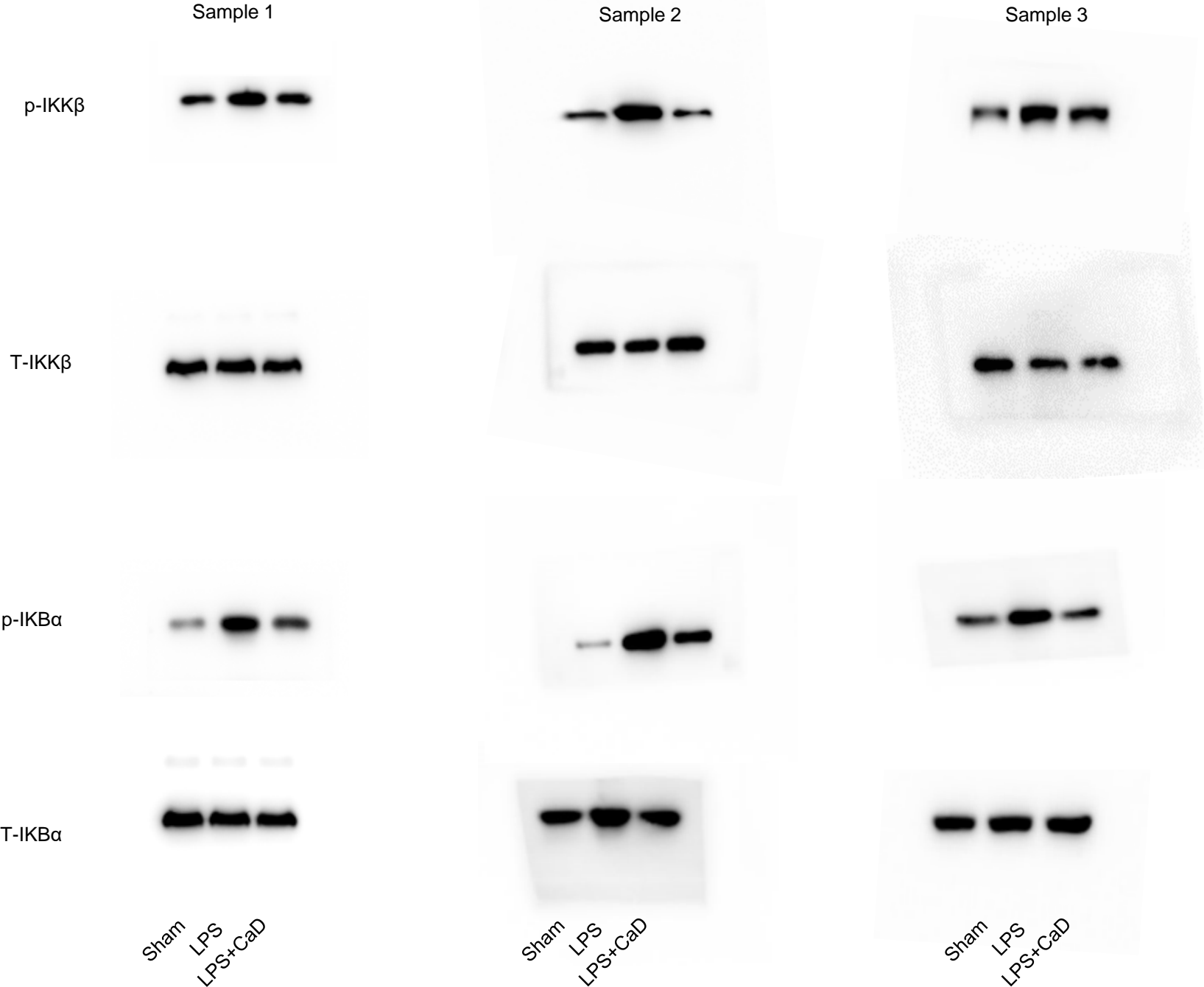

Fig.6A

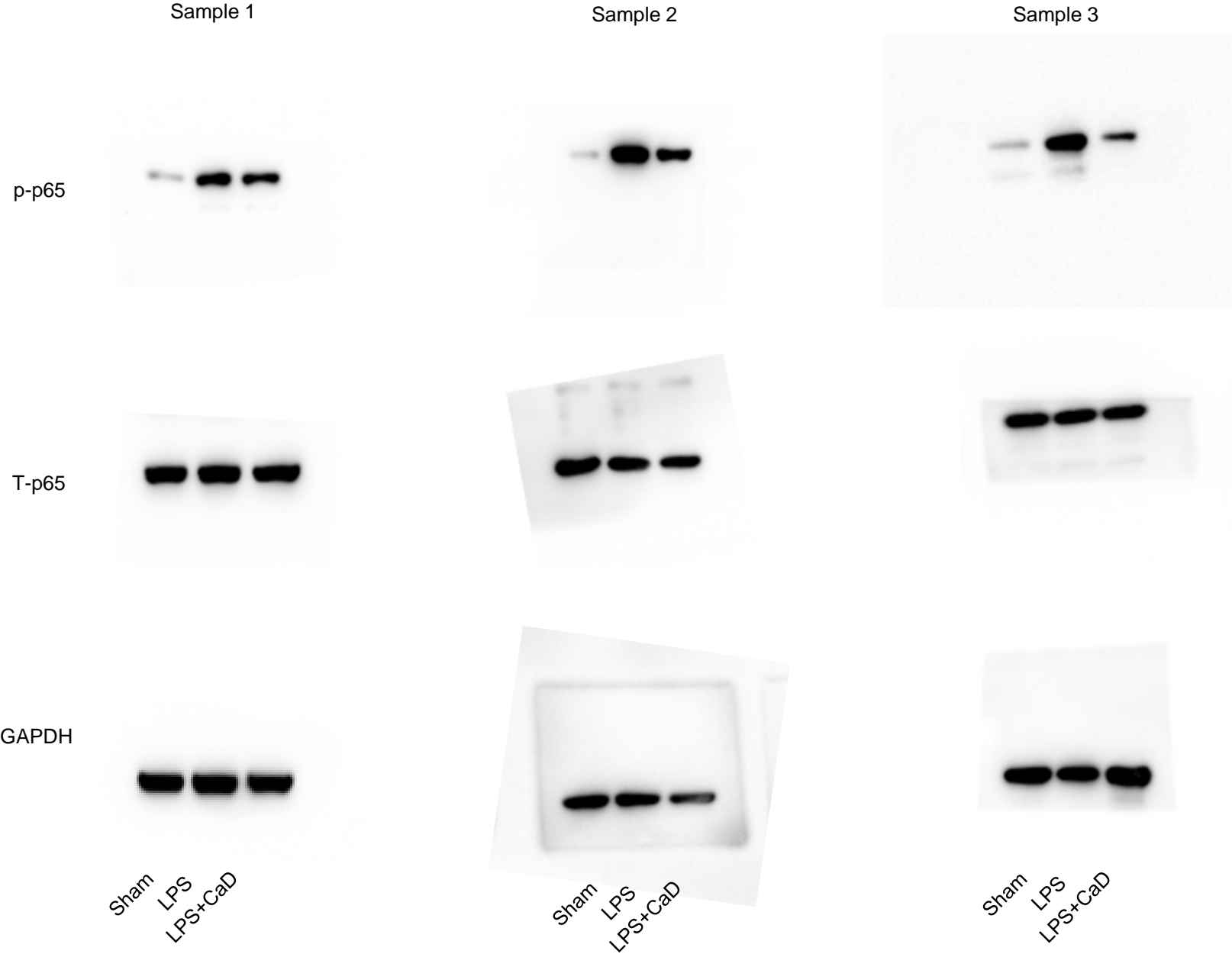

Fig6B

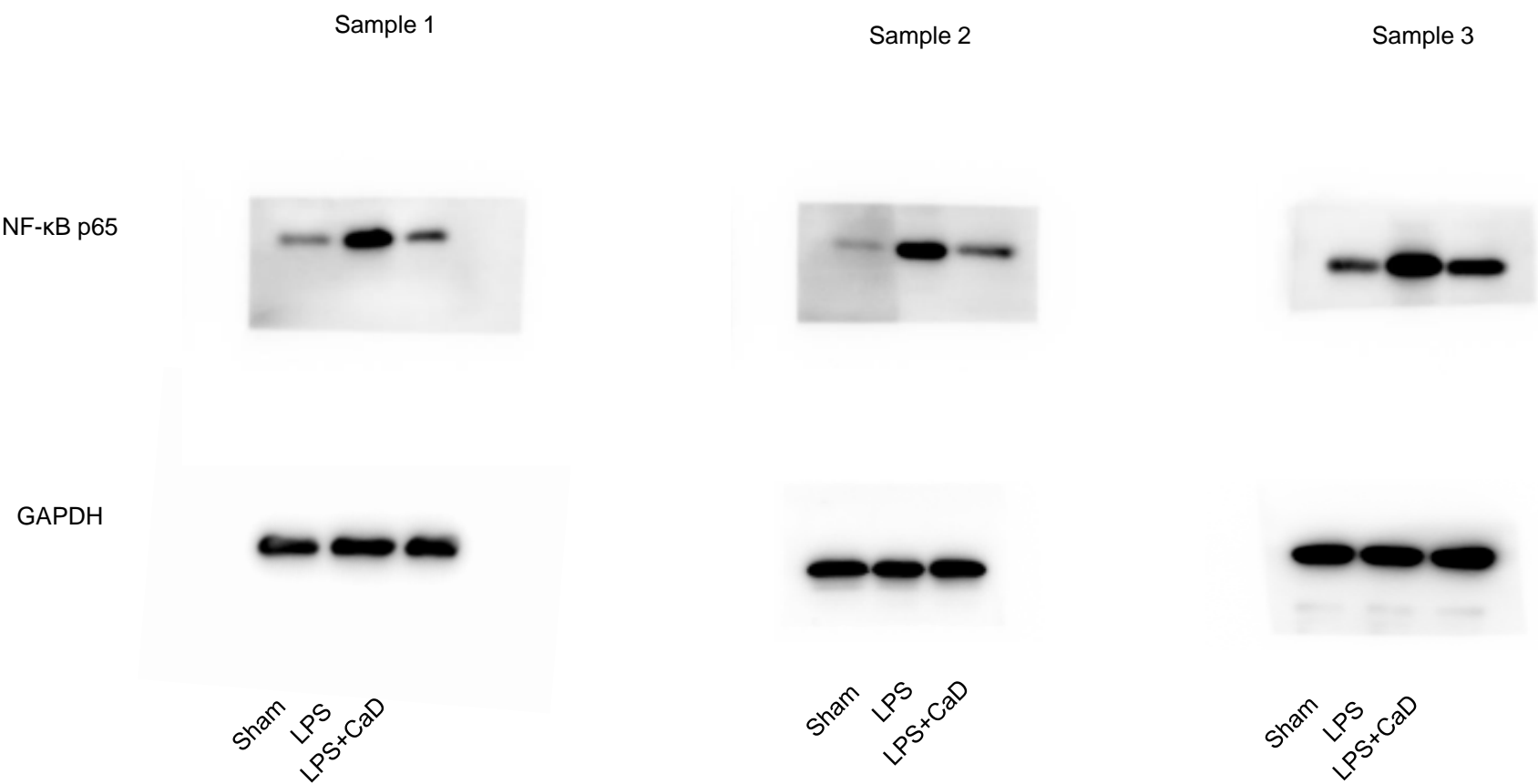

Supplement: Supplemental Material [file KBIE_A_2024394_SM8377.zip › supplementary/suppl.pdf]
